# Multiredox Polyoxovanadate‐Based Ionic Liquids for Nonaqueous Redox Flow Batteries

**DOI:** 10.1002/cssc.202502185

**Published:** 2026-02-22

**Authors:** Ke Wang, Stefan Repp, Moritz Remmers, Boris Mashtakov, Carsten Streb, Montaha Anjass

**Affiliations:** ^1^ Institute of Inorganic Chemistry Ulm University Ulm Germany; ^2^ Department of Chemistry Johannes Gutenberg University Mainz Mainz Germany; ^3^ Department of Chemistry University of Sharjah Sharjah UAE

**Keywords:** ionic liquids, multielectron transfers, polyoxovanadate, redox flow battery, solubility

## Abstract

Redox‐active ionic liquids (ILs) represent a promising class of energy carriers due to their intrinsic ionic conductivity, negligible volatility, and electron‐transfer capability. However, the design of ILs capable of reversible multielectron storage is still in its infancy. In this work, we report a family of mixed‐valence polyoxovanadate‐based ionic liquids (POV‐ILs) obtained by combining the highly redox‐active, mixed‐valence cluster (*n*Bu_4_N)_4_)_8_[V_14_O_34_Cl][(MgOH)V_13_O_33_Cl] with a series of bulky quaternary ammonium cations. Cation exchange transforms the solid precursor into liquid‐like POV‐ILs, dramatically enhancing solubility in organic solvents, such as acetonitrile, THF, and glymes, making them ideal compounds for nonaqueous redox flow batteries (NRFBs). Electrochemical studies demonstrate that these POV‐ILs retain the reversible multielectron redox activity of the parent cluster across a wide potential window, enabling their use as symmetric electrolytes in NRFBs. Flow‐cell demonstration confirms stable multielectron cycling, with electrolyte remixing mitigating capacity fading. By integrating the redox versatility of POVs with the solubility and processability of ILs, this work establishes a new design strategy for redox‐active electrolytes and highlights the promise of POV‐ILs for next‐generation, high‐energy‐density NRFBs.

## Graphical abstract

Here, we present a family of highly redox‐active polyoxovanadate‐based ionic liquids, that exhibit markedly enhanced solubility and multie‐lectron transfers in various nonaqueous media. We apply these mixed‐valence ionic liquids as redox‐active species in both catholyte and anolyte, and demonstrate symmetric nonaqueous redox flow batteries with stable operation over several days.

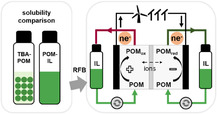

## 1 Introduction

Redox flow batteries (RFBs) are critical for sustainable energy schemes as they allow scalable, decoupled power generation and energy storage [1]. Their electrolyte solutions typically consist of redox‐active species and a supporting salt dissolved in a solvent [2]. The volumetric energy density of an RFB is governed by the number of electrons transferred, the operating cell voltage, and the effective concentrations of redox species. Additional factors that further limit the efficiency are ionic conductivity, reaction kinetics, and operating temperature. Aqueous electrolyte solutions offer favorable ionic conductivity and reaction kinetics; however, their operating temperature and cell voltage are constrained by the stability of water, while operation under acidic condition causes significant concerns regarding component corrosion [2]. In contrast, nonaqueous RFBs (NRFBs) benefit from various organic solvents (such as acetonitrile (MeCN) and propylene carbonate), which provide broad electrochemical stability windows [2, 3, 4, 5]. In addition, some organic solvents provide lower freezing points, which enable RFBs to operate in cold conditions [5]. However, in organic solvents, many redox‐active molecules tend to show low solubility or unfavorable redox stability, leading to low energy density or short life of NRFBs [6, 7, 8, 9].

In recent years, ionic liquids (ILs) have emerged as attractive materials in electrochemical energy applications, owing to intriguing features, such as negligible volatility, low flammability, and wide electrochemical stability window [10, 11]. ILs are salts that exhibit melting points below 100°C [10, 11, 12]. Many researchers focus on employing them as redox‐inactive supporting salts or solvents in electrolytes. In fact, ILs can serve as energy carriers in the RFB electrolytes if the cationic or anionic component is electroactive [13, 14, 15]. However, redox‐active ILs used for energy storage in the RFB operation often suffer from a limited number of electrons transferred and unfavorable reversibility [13, 14, 15]. Up to now, the design of ILs capable of reversible multielectron storage is still in its infancy.

One route to this end is the use of molecular metal oxide anions, so‐called polyoxometalates (POMs), as anionic IL component. POMs are molecular metal oxides based on high‐valent, early transition metals (often Mo, W, V), often exhibiting reversible multielectron storage capability and tunable redox properties [16, 17]. Pairing suitable bulky organic countercations with anionic POM ions can yield POM‐based ILs (POM‐ILs) [18, 19, 20, 21, 22, 23]. Combining the unique advantages of POM's multiredox capability and IL's effective ion dissociation, POM‐ILs can be promising high‐capacity electroactive materials for electrochemical energy storage systems.

Recently, POMs have been explored as redox‐active molecules for RFBs [23, 24, 25, 26, 27]. Cronin and coworkers developed an aqueous POM‐based RFB (POM–RFB) that achieved an energy density of 225 Wh L^−1^ by employing a Dawson‐type POM, Li_6_[P_2_W_18_O_62_], as anolyte and Br_2_/Br^‐^ redox couple as catholyte [25]. Ai et al. reported that an H_6_[P_2_W_18_O_62_] anolyte exhibited superior solubility compared with alkali‐ion counterparts, both at room temperature and low temperature of —20°C [26]. Using this POM‐based anolyte, low‐temperature asymmetric aqueous RFBs (ARFBs) were demonstrated. Furthermore, Stimming and coworkers employed distinct POM ions, [SiW_12_O_40_]^4—^ in the anolyte and [PV_14_O_42_]^9—^ in the catholyte, to construct an ARFB capable of 155 stable cycles over 14 days with supplied inert gas and chemical reducing agent [24]. Despite these advances, challenges remain. In POM‐based ARFBs, variation in electrolyte acidity can alter the pH‐dependent electron‐transfer behaviors of POM clusters, and HER (hydrogen evolution reaction) side reaction occurring at low reduction potentials can limit the device performance [23, 24, 25, 26, 27]. In POM‐based asymmetric ARFBs, one major concern is electrolyte contamination across the separator, especially when small‐sized redox species are employed in the electrolyte [23, 24, 25, 26, 27]. To mitigate this issue, Liu et al. reported a symmetric H_6_[CoW_12_O_40_]‐based ARFB with the central Co atom cycled between Co^III^ and Co^II^ in the catholyte, while tungsten (W) centers transited between W^V^ and W^IV^ in the anolyte [27]. However, the mismatch in the number of electrons transferred per molecule between the anolyte and catholyte induced electrolyte volume imbalance and osmotic pressure gradients, ultimately compromising operational stability.

The above issues can be, in principle, circumvented in nonaqueous electrolyte solutions. In fact, POM‐based symmetric NRFBs have been explored over the past decade [28, 29, 30, 31, 32, 33]. In a pioneering effort, a vanadium‐substituted (*n*Bu_4_N)_4_H_3_[SiV^V^_3_W^VI^_9_O_40_] cluster was used for a symmetric NRFB demonstration in propylene carbonate, but the loss of half initial capacity was observed after 10 charge–discharge cycles [28]. Barteau and coworkers demonstrated stable battery performance using Li_3_[PMo_12_O_40_] as active material; however, the redox processes were limited to one‐electron transfer on each side, with a small theoretical potential difference of 0.36 V [30]. In addition to these issues, POM clusters typically display lower solubility in nonaqueous media than in aqueous solutions, which can result in limited volumetric energy density [28]. To overcome this, Matson and coworkers covalently modified polyoxovanadate (POV) framework with alkoxide ligands, yielding POV–alkoxide clusters with enhanced solubility in acetonitrile (MeCN), but these clusters still require validation in practical RFB demonstration. Similarly, Peake et al. achieved two‐order‐magnitude solubility enhancement of a POM cluster, (*n*Bu_4_N)_3_[PW_11_O_39_(SiC_6_H_5_)_2_O], by organofunctionalization of the parent [PW_12_O_40_]^3–^ cluster [33], Nonetheless, such structural modifications raise concerns about unexpected alterations of redox behaviors since the functionalized frameworks differ significantly from the pristine ones [34, 35]. Thus, the POM clusters enabling both high effective concentrations and multiredox transfers under a wide potential window in nonaqueous media can be intriguing candidates for RFB systems.

Since countercations play an essential role in controlling the solubility of POM clusters in solutions, pairing bulky countercations with POM anions can be an effective approach to achieve solubility enhancement. Highly redox‐active cocrystal POV clusters, (*n*Bu_4_N)_4_)_8_[V_14_O_34_Cl][(MgOH)V_13_O_33_Cl] (denoted as TBA‐**{MV**_**13**_ **}**, where **M** corresponds to V=O or MgOH), which have been recently reported [36], can offer 14 redox waves in a wide potential range from −2.15 V to + 1.35 V (vs. Fc^+^/Fc). Bulk electrolysis was also utilized, determining a total of 16 electron transfers in these redox waves, which were tentatively assigned to 12 single‐electron transfers and two two‐electron transfers, respectively. In this work, by pairing the anionic components of the POV clusters with a series of bulky quaternary ammonium cations, we describe a family of POV‐ILs with significantly enhanced solubility and multiredox capability in organic solvents, which are widely used in electrolyte engineering in battery systems. This implies the unique potential of redox‐active POV‐ILs in energy storage applications. Symmetric RFB demonstrations with stable charge–discharge performance indicate that the mixed‐valence POV‐ILs can be promising redox materials for RFB electrolyte systems.

## 2 Results and Discussion

**FIGURE 1:**
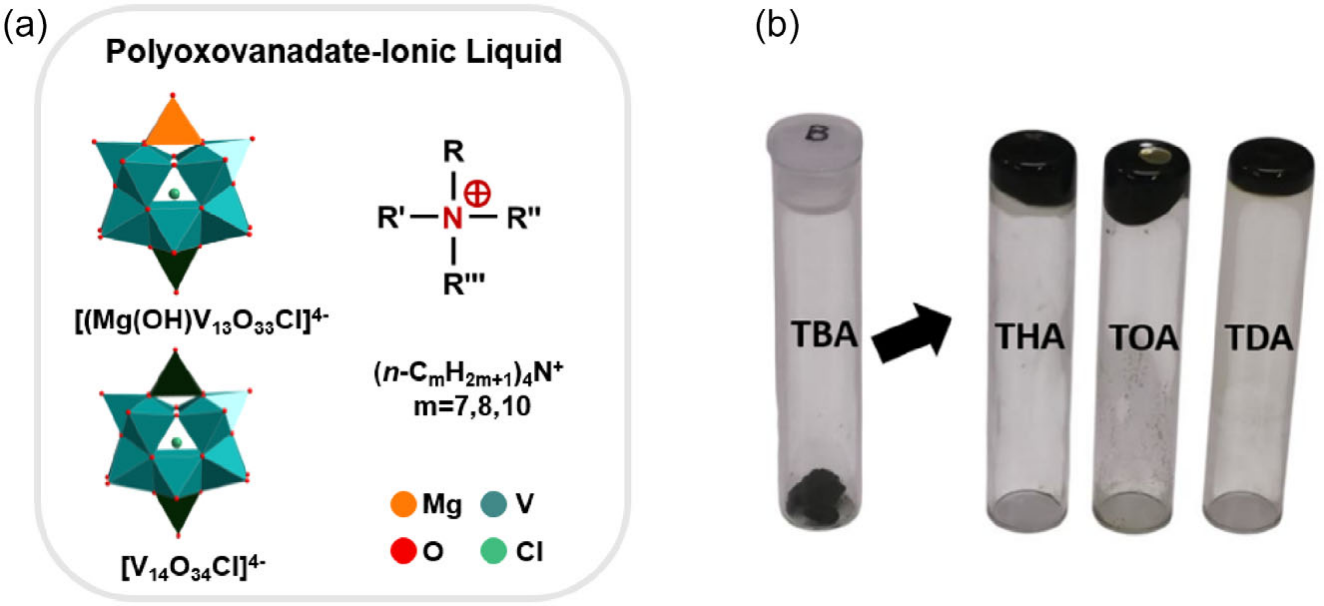
(a) POV‐ILs composed of bulky tetraalkylammonium cations and anionic vanadates formed for the cocrystal TBA‐**{MV**_**13**_ **}** clusters; (b) photograph of TBA‐**{MV**_**13**_ **},** THA‐**{MV**_**13**_ **},** TOA‐**{MV**_**13**_ **}**, and TDA‐**{MV**_**13**_ **}** at ambient temperature.

A series of long‐chain quaternary alkylammonium cations, including THA^+^ (tetraheptylammonium), TOA^+^ (tetraoctylammonium), and TDA^+^ (tetradecylammonium), were chosen to pair the anionic components of the parent TBA‐**{MV**_**13**_ **}** clusters, yielding THA‐**{MV**_**13**_ **}**, TOA‐**{MV**_**13**_ **}**, and TDA‐**{MV**_**13**_ **}**, respectively. At ambient temperature, the dark green TBA‐**{MV**_**13**_ **}** cluster is in solid state, while THA‐**{MV**_**13**_ **}**, TOA‐**{MV**_**13**_ **},** and TDA‐**{MV**_**13**_ **}** exhibited viscous liquid‐like states and retained the dark green color (see Figure 1) [36]. Differential scanning calorimetry (DSC) analysis showed that the melting points of the three POV clusters after cation exchange were well below 100°C, confirming the formation of IL phases (see Figure S1 and Table S1).The countercation exchange was based on earlier literature reports [18, 19, 21, 22] and characterized by various techniques as follows. For the IL clusters, ATR‐FTIR spectroscopy exhibited the intensity of the band at ≈1462 cm^−1^, corresponding to CH_2_ scissoring bending, increased and the bands vibrations from CH_2_ group at 3000–2750 cm^−1^ had a red shift, which were probably caused by higher arrangement order of alkyl chains and increased van der Waals interactions, indicating the increase of the alkyl chain length of the quaternary ammonium cations (see Figure S2) [37]. The purity of the cation exchange was specified by proton NMR (see Figures S3–S6). The tetraalkylammonium cations can be observed in four peaks in the NMR spectroscopy. The only signal, which changed by using higher alkyl chains, was the peak at 1.3 ppm, which indicated no (*n*Bu_4_N)^+^ impurities in the IL phases and verified successful complete countercation exchange. Furthermore, cation exchange was confirmed using thermogravimetric analysis (TGA) in the range from 25°C to 700°C, which showed weight loss of 42 wt.‐% (calcd.: 43 wt.‐%), 57 wt.‐% (calcd.: 56.2 wt.‐%), 59 wt.‐% (calcd.: 59.3 wt.‐%), and 64 wt.‐% (calcd.: 64.4 wt.‐%) for TBA‐**{MV**_**13**_ **}**, THA‐**{MV**_**13**_ **}**, TOA‐**{MV**_**13**_ **}**, and TDA‐**{MV**_**13**_ **}**, respectively, corresponding to the decomposition of the organic countercations in relevant cluster (see Figure S7). UV–Vis spectroscopy revealed the retention of the two characteristic signals, which corresponded to ligand‐to‐metal charge transfer in the UV region peaking at ∼330 nm and intervalence charge transfer NIR region peaking at ∼950 nm, respectively, verifying the retention of mixed‐valence oxidation states of the POV clusters after conversion into IL phases (see Figure S8).

Due to the high viscosity of **{MV**_**13**_ **}**‐ILs at ambient temperatures, adding solvent into the redox‐active ILs is required for the application to RFB electrolyte systems. MeCN is one frequently used solvent in nonaqueous electrochemistry research, due to its low viscosity, high dielectric constant, and desirable electrochemical stability window [2]. The solubility of TBA‐**{MV**_**13**_ **}** and **{MV**_**13**_ **}**‐ILs in MeCN was investigated at room temperature. The solubility of TBA‐**{MV**_**13**_ **}** in MeCN was less than 30 mM since there were still obvious undissolved precipitates after vigorous magnetic stirring overnight. By comparison, 60 mM THA‐**{MV**_**13**_ **}** and 60 mM TOA‐**{MV**_**13**_ **}** can be fully dissolved in MeCN, and the resulting solution phases showed low‐viscosity fluidic liquid behavior, which was suitable to be used as redox‐active electrolytes for all‐liquid RFB systems (see Figure S9). In contrast, the addition of 60 mM TDA‐**{MV**_**13**_ **}** in MeCN yielded a flowable but viscous mud‐like mixture. The UV–Vis test showed that the solubility of TOA‐**{MV**_**13**_ **}** in MeCN was approximately 130 mM, which was significantly higher than that of TBA‐**{MV**_**13**_ **}**. However, the solubility of TDA‐**{MV**_**13**_ **}** was only 30 mM (see Figure S10). In addition to MeCN, the solubilizing capability of TBA‐**{MV**_**13**_ **}** and **{MV**_**13**_ **}**‐ILs in some other organic solvents was compared, including tetrahydrofuran (THF), tetraethylene glycol dimethyl ether (TEGDME), diglyme, and dichloromethane (DCM) (see Figure S11). TBA‐**{MV**_**13**_ **}** is hardly soluble in THF, TGEDME, and diglyme, while the three **{MV**_**13**_ **}**‐ILs can be easily soluble, except that THA‐**{MV**_**13**_ **}**‐IL is comparatively less soluble in TEGDME. In terms of DCM, after vigorously stirring, TBA‐**{MV**_**13**_ **}** was slightly soluble, while **{MV**_**13**_ **}**‐ILs were completely soluble and give dark green color. The whole solubility tests indicated the influence of alkyl chain length on the solubility of POMs‐IL and corroborated that POM‐IL formation can be a propitious approach for solubility enhancement.

**FIGURE 2:**
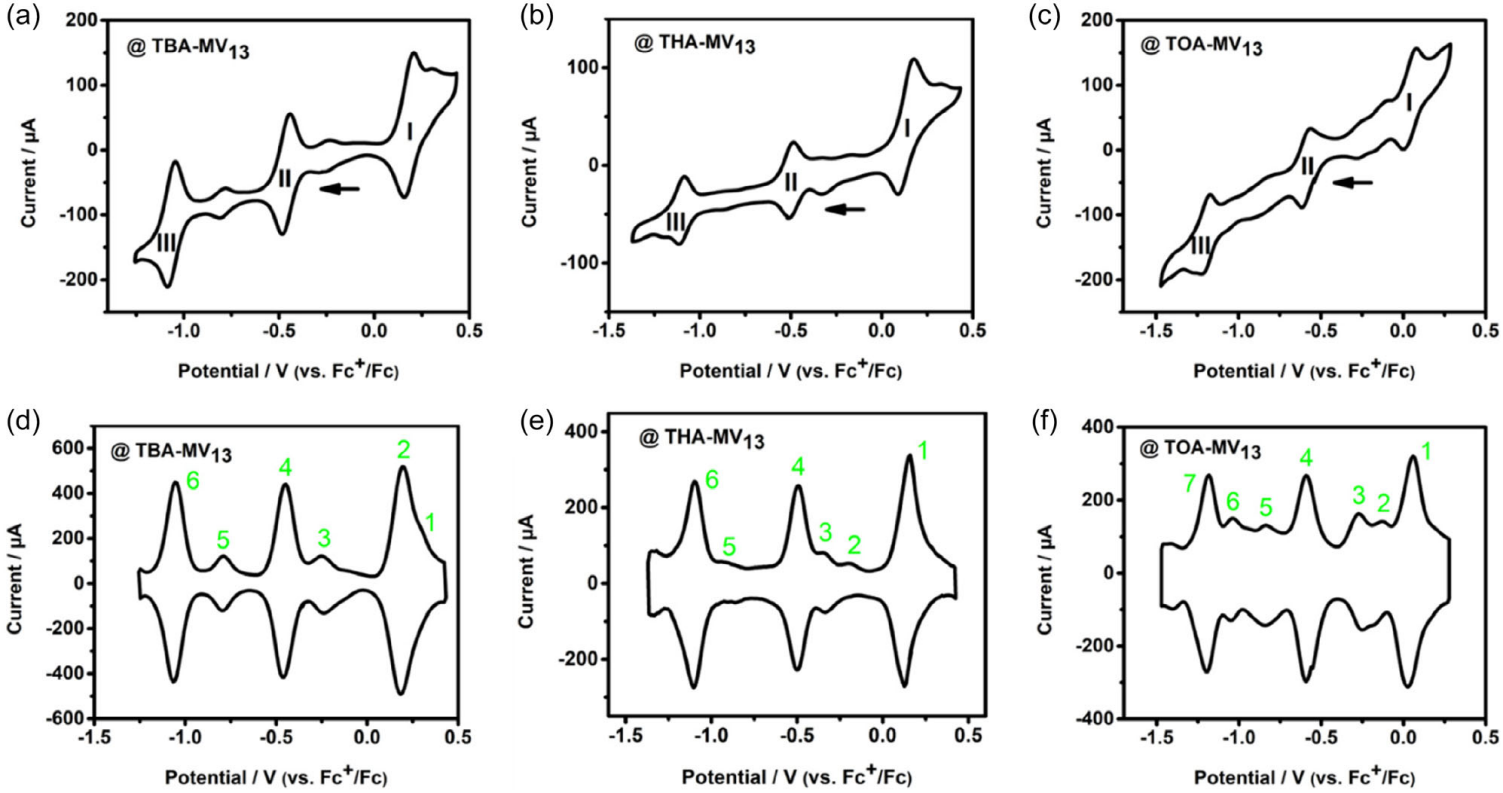
Cyclic voltammograms of (a) 0.5 mM TBA‐**{MV**_**13**_ **}** with 0.1 M TBAPF_6_ in MeCN, (b) 0.5 mM THA‐**{MV**_**13**_ **}** with 0.1 M THABF_4_ in MeCN, and (c) 0.5 mM TOA‐M**V**_**13**_ with 0.1 M TOABF_4_ in MeCN, respectively. Square‐wave voltammograms of (d) 0.5 mM TBA‐**{MV**_**13**_ **}** with 0.1 M TBAPF_6_ in MeCN, (e) 0.5 mM THA‐**{MV**_**13**_ **}** with 0.1 M THABF_4_ in MeCN, and (f) 0.5 mM TOA‐**{MV**_**13**_ **}** with 0.1 M TOABF_4_ in MeCN, respectively. Carbon felt (5 mm × 5 mm × 6.5 mm) is used as the working electrode. Scan rate is 2 mV s^−1^.

The electrochemical behaviors of the TBA‐**{MV**_**13**_ **}** and **{MV**_**13**_ **}**‐ILs were tested in three‐electrode setup, where carbon felt is used as working electrode, with platinum wire counter electrode, and silver wire in a glass frit containing electrolyte solution as quasi‐reference electrode. Carbon‐based materials, such as carbon felt and carbon paper, are most frequently used electrode materials in flow battery applications, due to the advantages of high specific surface area, low electric resistance, and robust (electro‐)chemical stability. Cyclic voltammetry (CV) and square‐wave voltammetry (SWV) techniques were employed to compare the redox behaviors of the parent TBA‐**{MV**_**13**_ **}** cluster and the **{MV**_**13**_ **}**‐ILs on carbon felt and glassy carbon electrode, respectively. In the reported electrochemical studies on redox‐active POM‐ILs, TBAPF_6_ and TEABF_4_ (tetraethylammonium tetrafluoroborate) were often used as supporting electrolytes, and the concentration of the supporting electrolyte was much larger than that of the redox molecule in the tested solutions [38, 39]. As a result, cation exchange between the tested POM‐IL and the supporting salt can possibly occur, which can influence the analysis on the true redox behaviors of POM‐ILs in solutions. In addition, the charge‐compensating TBA^+^ or TEA^+^ cation can interact with the anionic component of the tested POM‐IL during the electrochemical reduction process, which can possibly cause the formation of precipitates. Here, TBAPF_6_ and TOABF_4_ were used as supporting electrolytes for the CV/SWV measurements of TBA‐**{MV**_**13**_ **}** and TOA‐**{MV**_**13**_ **}** in MeCN, respectively. Since there are no commercially available tetraheptylammonium and tetradecylammonium salts suitable as supporting electrolytes for electrochemical tests, tetrahexylammonium salt (THABF_4_) was chosen as supporting electrolyte for THA‐**{MV**_**13**_ **}** in CV/SWV test, while the CV/SWV tests on TDA‐**{MV**_**13**_ **}** were not given. The electrochemical performance of the native mixed‐valence cluster TBA‐**{MV**_**13**_ **}** has been reported previously and revealed an exceptionally rich multielectron redox chemistry [36]. In acetonitrile, TBA‐**{MV**_**13**_ **}** exhibits up to 14 distinct redox transitions on a glassy carbon electrode (Ø = 3 mm). In the present study, the electrochemical response of TBA‐**{MV**_**13**_ **}** is also investigated on carbon felt electrodes under conditions relevant to flow battery operation (see Figure 2a,d). While the same underlying multiredox behavior is retained, three dominant redox events (denoted events I–III from high potential to low potential) are clearly resolved within the potential range from −1.30 to 0.50 V vs. Fc^+^/Fc. Redox event I was positive to “open circuit potential” (OCP), while redox events II and III were negative to it. Additional small redox features can still be observed which might be attributed to partially resolved one‐electron steps associated with the multiple redox‐active V centers in the cocrystallized mixed‐valence clusters. On carbon felt (much higher surface area), mass transport kinetics and the porous electrode geometry may cause closely spaced redox waves to merge into broader composite redox waves, thereby reducing the apparent number of resolved transitions compared to the glassy carbon [36]. The differences in the types of electrode surfaces and mass transport kinetics can lead to varied redox waves [40].

Following countercation exchange, the mixed‐valence THA‐**{MV**_**13**_ **}** and TOA‐**{MV**_**13**_ **}** ILs show similar reduction and oxidation processes to TBA‐**{MV**_**13**_ **}** on carbon felt at the corresponding potentials (E_1/2_) in a potential range from −1.30 V to 0.50 V (vs. Fc^+^/Fc) (see Figure 2b–f), again showing three dominant redox events (I–III). This confirms that the **{MV**_**13**_ **}**‐ILs can retain the multiredox activity in the MeCN‐based solutions after cation exchange. TOA‐**{MV**_**13**_ **}** also provided similar redox waves to TBA‐**{MV**_**13**_ **}** on glassy carbon electrode (Ø = 3 mm, see later section) [36], again the number of the redox waves on glassy carbon was higher than on carbon felt. The reversible multiredox capability in the CV/SWV analysis indicated the potential of **{MV**_**13**_ **}**‐ILs as energy carriers in NRFB electrolyte systems.

**FIGURE 3:**
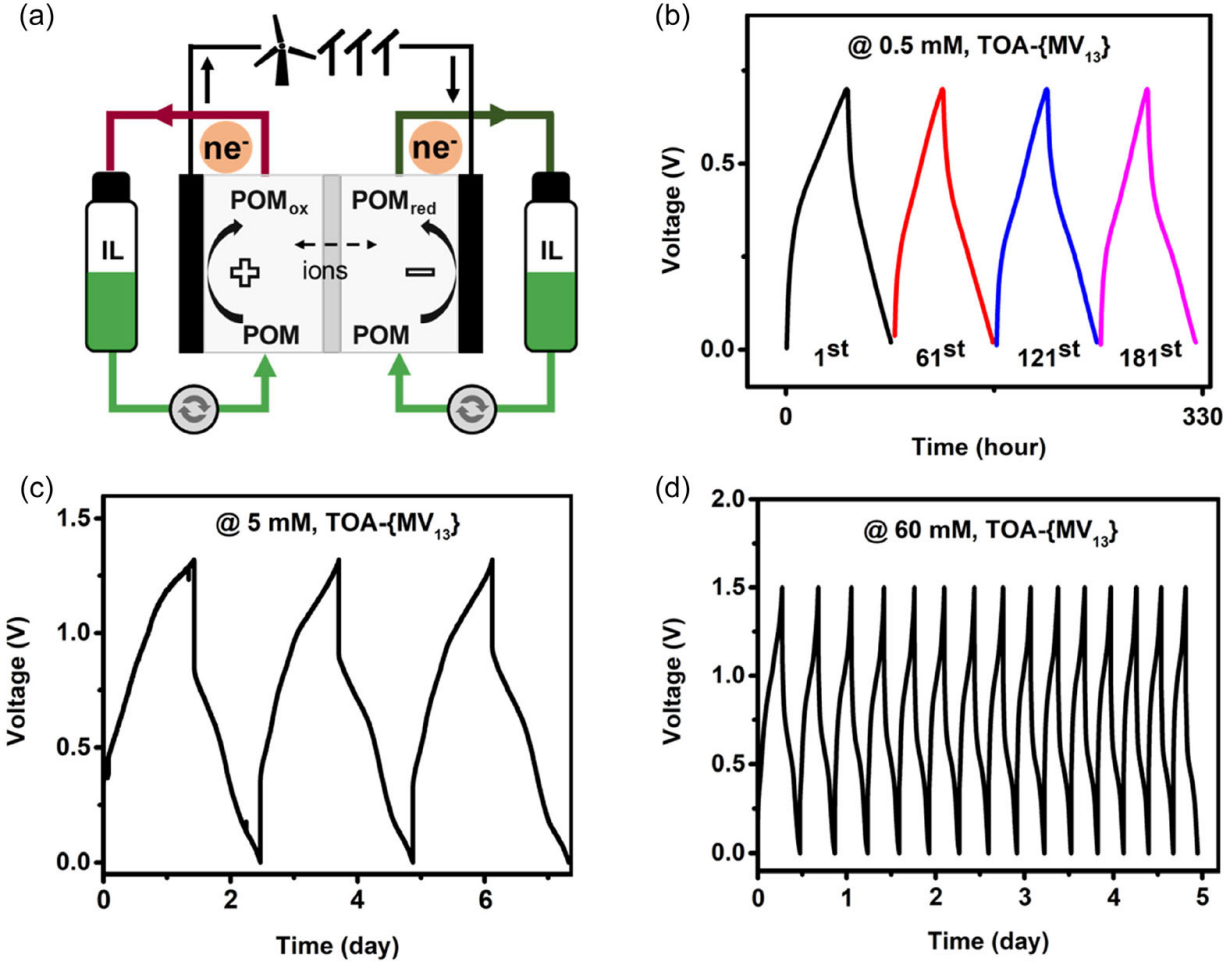
(a) Schematic illustration of a symmetric POM‐IL‐based RFB, (b) voltage files of a symmetric TOA‐**{MV**_**13**_ **}** RFB employing 0.5 mM TOA‐**{MV**_**13**_ **}** at flow mode, 12 ml of electrolyte at each side, (c) voltage files of a symmetric TOA‐**{MV**_**13**_ **}** RFB employing 5 mM TOA‐**{MV**_**13**_ **}** at flow mode, 10 ml of electrolyte at each side, and (d) voltage files of a symmetric TOA‐**{MV**_**13**_ **}** RFB employing 60 mM TOA‐**{MV**_**13**_ **}** at static mode, 0.25 ml of electrolyte at each side.

The **{MV**_**13**_ **}**‐ILs are very attractive redox materials in nonaqueous electrolyte solutions due to reversible multiredox processes in a wide potential range and high effective concentrations in various organic solvents. The redox events in both low and high redox potentials indicate that **{MV**_**13**_ **}**‐ILs can be applied as electrolyte materials in symmetric RFBs (see Figure 3a), in which the initial catholyte and anolyte are the same.

**FIGURE 4:**
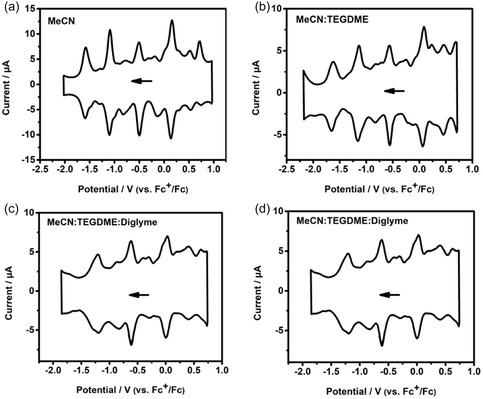
Square‐wave voltammograms of 0.5 mM TOA‐**{MV**_**13**_ **}** with 0.1 M TOABF_4_ in (a) MeCN, (b) MeCN/TEGDME solvent mixture (1:1, v/v), (c) MeCN/diglyme solvent mixture (1:1, v/v), (d) MeCN/TEGDME/diglyme solvent mixture (2:1:1, v/v/v), respectively. Glassy carbon (Ø = 3 mm) is used as the working electrode. Scan rate is 100 mV s^−1^.

In this work, the charge–discharge performance of symmetric NRFBs using multiredox **{MV**_**13**_ **}**‐ILs in MeCN‐based solutions was investigated at different concentrations in a lab‐scale RFB in an argon‐filled glove box. In an initial cell test, low concentration of 0.5 mM **{MV**_**13**_ **}**‐ILs was used for both half cells, and carbon felts were used as electrode materials. The two half‐cells were separated by two pieces of microporous separators (Celgard 2320), and 12 ml of electrolyte solution was used for each half cell. The flow cells were galvanostatically charged and discharged at 0.04 mA cm^−2^ and 0.02 mA cm^−2^, respectively, with cutoff charging voltage of 0.7 V and discharging voltage of 0.02 V, respectively. The symmetric TOA‐**{MV**_**13**_ **}‐**based RFB was operated for 240 charge–discharge cycles (see Figure 4b and Figure S12). Only one voltage plateau appeared, which correlated well with the electrochemical investigation of TOA‐**{MV**_**13**_ **}** on carbon felt. In the first 60 charge–discharge cycles, Coulombic efficiency (CE) was 60–70%, which was close to the CE values in other NRFB work also using porous Celgard separators [9, 41]. Since the compositions and concentrations of the catholyte and anolyte are identical, a facile electrolyte‐remixing approach can be adopted to alleviate capacity decay. After a period of battery operation, the catholyte and anolyte were mixed for electrolyte “rebalancing” to recover initial valence state of electrolytes; afterward, the mixed solution was divided equally and returned back into the positive and negative tanks, respectively, for further RFB operation. The electrolyte‐remixing treatment was performed after every 60 charge–discharge cycles. Capacity decay was effectively alleviated after the electrolyte‐remixing treatment (see voltage files of the 1^st^ cycle, 61^st^ cycle, 121^st^ cycle, 181^st^ cycle in Figure 3b). These analogous voltage files implied good chemical stability of **{MV**_**13**_ **}** clusters in the electrolyte solution during the battery operation. In addition, the symmetric THA‐**{MV**_**13**_ **}** RFB with 0.5 mM THA‐**{MV**_**13**_ **}** was operated for 160 cycles with the electrolyte‐remixing treatment (see Figure S13).

Anion exchange membranes are cost‐effective and only need simple pretreatment, and have been explored recently in NRFBs [41]. AMI‐7001 was reported to provide low permeability of POM anions and allow the transferring anions, such as PF_6_^−^, to across the separator for charge balance during the battery operation [31, 42]. In the subsequent charge–discharge testing of 5 mM of TOA‐**{MV**_**13**_ **}** in the symmetric RFB, a piece of AMI‐7001 membrane was used as the separator, which was pretreated in the 0.1 M TOABF_4_/MeCN solution for 1 day. A higher charging voltage of 1.32 V was used for a deeper utilization of the TOA‐**{MV**_**13**_ **}**. The voltage was obtained by charging the battery with 99% of the theoretical capacity (based on four‐electron transfers). The charging capacity can be determined by the Faraday equation, *Q* = *z* × *n* × *F*, where *Q* is charging capacity, *z* is number of electron transfer, *n* is mole of reactive species, and *F* is Faraday constant. The capacity, corresponding to four‐electron storage of the polyoxovanadate clusters in the battery electrolyte solutions, is *Q* = 4 × 10 mL × 5 mM × 26.8 Ah/L = 5.36 mAh. The first charging step of the battery operation was finished when the charging capacity reached to 5.3 mAh (5.3/5.36 mAh ≈ 99%). The corresponding charging voltage (1.32 V) obtained was used as cutoff condition for the following cycles. The voltage files showed more voltage plateaus in the charging processes, which indicated more electron transferred in this battery test (see Figure 3c). In addition, the high similarity of the voltage files implied the stability of TOA‐**{MV**_**13**_ **}** under the wider operating voltage. CE in the first cycle was 73.0%, and increased to 96.3%, maybe due to “membrane activation,” in the third cycle, in which the voltage efficiency and energy efficiency were 51.0% and 49.2%, respectively.

Finally, a higher concentration of TOA‐**{MV**_**13**_ **}** electrolyte up to 60 mM was assessed in the RFB with carbon paper as electrode materials. The battery was run in static mode at the charging voltage of 1.5 V. The stable charge–discharge performance approved the feasibility of high‐concentration POM‐ILs electrolytes applied into RFB systems (see Figure 3d). CE in the first cycle was 73.5%, and increased to 96.5% in the third cycle, in which the voltage efficiency and energy efficiency were 52.8% and 50.9%, respectively. The CE was kept stable in the following cycles.

In addition to single‐solvent‐based electrolyte, hybrid electrolyte engineering is an attractive strategy to improve electrolyte properties, such as viscosity, ionic conductivity, and electron‐transfer kinetics, but this requires solubility capability of redox‐active molecules in various solvents [7, 43, 44]. Glyme‐based solvents exhibit low viscosity but low dielectric constant, such as DME (µ, 0.46 mPa s; *ε*, 7.2) and diglyme (µ, 0.99 mPa s; *ε*, 7.23), while carbonate‐based solvents offer high dielectric constant but high viscosity, such as propylene carbonate (µ, 2.53 mPa s; *ε*, 64.9) [2]. **{MV**_**13**_ **}**‐ILs enable high solubility in a myriad of organic solvents, including TEGDME and diglyme, in which the parent TBA‐**{MV**_**13**_ **}** clusters are hardly soluble. The redox behaviors of TOA‐**{MV**_**13**_ **}** in pure MeCN and in binary solvent mixtures (MeCN/diglyme, MeCN/TEGDME) and ternary solvent mixture (MeCN/TEGDME/diglyme) on glassy carbon (Ø = 3 mm) were examined (see Figure 4). In the solvent mixtures, the volume ratio of MeCN was kept at 50%. TOA‐**{MV**_**13**_ **}** underwent several redox transitions in the pure MeCN solvent in the potential range between −1.75 and 1.0 V, closely matching the redox profile of the native TBA‐**{MV**_**13**_ **}** cluster, thus indicating that the intrinsic multiredox activity of the parent framework is largely retained after cation exchange [36]. In the binary MeCN/TEGDME mixture, TOA‐**{MV**_**13**_ **}** can still exhibit 13 redox waves, although the redox behaviors became slightly different. In comparison, less redox events of TOA‐**{MV**_**13**_ **}** in the binary MeCN/diglyme mixture were observed. In the ternary MeCN/TEGDME/diglyme mixture, TOA‐**{MV**_**13**_ **}** can provide 10 redox transitions in the potential range. These results showed solvents that can influence the redox behaviors of TOA‐**{MV**_**13**_ **}**, and TOA‐**{MV**_**13**_ **}** can still maintain the multiredox capability in hybrid electrolyte solutions.

Notably, during NRFB operation, the volatile property of organic solvents can cause solvent loss in electrolytes, which can affect the fluidity of POM‐IL electrolytes, especially in the case of high concentration. In addition, since most organic solvents are toxic and flammable, the use of NRFBs brings about safety concerns. Mixing a suitable inert IL serving as both SE and solvent with the redox‐active POM‐IL might be an approach to provide both negligible volatility and nonflammability [45, 46].

## 3 Conclusion

In summary, we introduce mixed‐valence polyoxovanadate‐based ionic liquids as a new class of multielectron redox‐active electrolytes. Pairing bulky alkylammonium cations with POV anions not only preserves their multielectron redox activity but also enhances solubility in various organic solvents, in which the parent solid‐state counterpart is hardly soluble. Electrochemical studies and symmetric flow‐battery tests demonstrate reversible multielectron storage capability in MeCN‐based electrolyte solutions with stable cycling aided by facial electrolyte‐remixing technique. The retention of redox functionality in binary and ternary solvent‐based electrolyte solutions indicates the potential advantage of POV‐ILs in hybrid electrolyte engineering. Beyond electrolyte development, further improvement in electrode materials with high electrocatalytic activity for POM clusters in electrolyte solutions, as well as separators with high selectivity and low resistance, is also required to fully exploit the high energy density of these electrolytes. With their tunable cation–anion combinations, redox‐active POV‐ILs provide a versatile platform for advancing NRFBs toward higher energy density of longer operational lifetimes.

## Author Contributions

**Ke Wang:** conceptualization, development of the methodology, validation, formal analysis, writing – the original draft (lead), data curation, writing – review & editing, investigation, visualization. **Stefan** **Repp**: development of the methodology, formal analysis, and writing – review & editing; **Moritz** **Remmers:** development of the methodology, characterization such as NMR, UV‐vis, formal analysis, and writing – review & editing;and **Boris** **Mashtakov**: carried out the synthesis and characterization such as NMR, UV–Vis, formal analysis, and writing – review & editing; **Montaha** **Anjass** and **Carsten** **Streb:** conceptualization, resources, writing – the original draft (supporting), writing – review & editing, supervision, project administration, funding acquisition. All authors have approved the final version of the article.

## Supporting Information

Additional supporting information can be found online in the Supporting Information section. **Supporting** **F** **ig.** **S1:** DSC analysis of (a) TBA‐{MV13}, (b) THA‐{MV13}, (c) TOA‐{MV13}, and (d) TDA‐{MV13}, under N2 atmosphere with the heating rate of 10.0 K min^−1^. **Supporting Fig.** **S2:** ATR‐FTIR spectra of TBA‐{MV13}, THA‐{MV13}, TOA‐{MV13}, TDA‐{MV13} normalized transmission. **Supporting Fig.** **S3:** ^1^H‐NMR of TBA‐{MV13}. Conditions: solvent: DMSO‐d6, 400 MHz, 16 scans. Signal assignments: *δ* (ppm) = 3.19 (m, 2 H, 1 x CH2, *n*Butyl4N+); 2.50 (s, DMSO); 2.08 (impurity); 1.98 (impurity); 1.90 (impurity); 1.57 (m, 2 H, 1 x CH2, *n*Butyl4N+); 1.32 (m, 2 H, 1 x CH2, *n*Butyl4N+); 1.10 (impurity); 0.93 (m, 3 H, 1 x CH3, *n*Butyl4N+). **Supporting Fig.** **S4:** 1H‐NMR of THA‐{MV13}. Conditions: solvent: DMSO‐d6, 400 MHz, 16 scans. Signal assignments: *δ* (ppm) = 3.17 (m, 2 H, 1 x CH2, *n*Heptyl4N+); 2.50 (s, DMSO); 2.08 (impurity); 1.98 (impurity); 1.90 (impurity); 1.57 (m, 2 H, 1 x CH2, *n*Heptyl4N+); 1.27 (m, 8 H, 4 x CH2, *n*Heptyl4N+); 1.10 (impurity); 0.93 (m, 3 H, 1 x CH3, *n*Heptyl4N+). **Supporting Fig.** **S5:** 1H‐NMR of TOA‐{MV13}. Conditions: solvent: DMSO‐d6, 400 MHz, 16 scans. Signal assignments: *δ* (ppm) = 3.33 (water); 3.17(m, 2 H, 1 x CH2, *n*Octyl4N+); 2.50 (s, DMSO); 2.08 (impurity); 1.99 (impurity); 1.91 (impurity); 1.57 (m, 2 H, 1 x CH2, *n*Octyl4N+); 1.27 (m, 10 H, 5 x CH2, *n*Octyl4N+); 1.10 (impurity); 0.87 (m, 3 H, 1 x CH3, *n*Octyl4N+. **Supporting** **Fig.** **S6:** 1H‐NMR of TDA‐{MV13}. Conditions: solvent: DMSO‐d6, 400 MHz, 16 scans. Signal assignments: *δ* (ppm) = 3.37 (water); 3.18(m, 2 H, 1 x CH2, *n*Decyl4N+); 2.50 (s, DMSO); 2.08 (impurity); 1.98 (impurity); 1.90 (impurity); 1.56 (m, 2 H, 1 x CH2, *n*Decyl4N+); 1.25 (m, 14 H, 7 x CH2, *n*Decyl4N+); 1.10 (impurity); 1.01 (impurity); 0.86 (m, 3 H, 1 x CH3, *n*Decyl4N+). **Supporting** **Fig.** **S7:** TGA tests of TBA‐{MV13}, THA‐{MV13}, TOA‐{MV13}, TDA‐{MV13}, respectively, at a heating rate of 10.0 K min^‐1^ in a range between 25°C and 700°C under O2/N2 which showed weight loss of 42 wt.‐% (calcd.: 43 wt.‐%), 57 wt.‐% (calcd.: 56.2 wt.‐%), 59 wt.‐% (calcd.: 59.3 wt.‐%), and 64 wt.‐% (calcd.: 64.4 wt.‐%) for TBA‐{MV13}, THA‐{MV13}, TOA‐{MV13}, TDA‐{MV13}, respectively. **Supporting Fig.** **S8:** UV–Vis spectra of (a) 0.025 mMol TBA‐{MV13}, (b) 0.025 mMol THA‐{MV13}, (c) 0.025 mMol TOA‐{MV13}, (d) 0.025 mMol TDA‐{MV13}. **Supporting Fig.** **S9:** (a) 30 mM TBA‐{MV13} in MeCN, 60 mM THA‐{MV13} in MeCN, 60 mM TOA‐{MV13} in MeCN, 60 mM TDA‐{MV13} in MeCN, respectively; (b) approximate 130 mM TOA‐{MV13} in MeCN. **Supporting Fig.** **S10:** (a) Absorbance spectra of TOA‐{MV13} in MeCN; (b) Beer–Lambert plot of absorbance of TOA‐{MV13} in MeCN at 221 nm; (c) absorbance spectra of TDA‐{MV13} in MeCN; (d) Beer–Lambert plot of absorbance of TDA‐{MV13} in MeCN at 221 nm. **Supporting Fig.** **S11:** The digital photographs of TBA‐{MV13} and {MV13}‐ILs in (a) THF; (b) TEGDME; (c) Diglyme; (d) DCM. **Supporting** **Fig.** **S12:** Cycling performance of a symmetric RFB using 0.5 mM TOA‐{MV13} in 12 ml MeCN with 0.1 M THABF4 at each side. **Supporting Fig.** **S13:** Cycling performance of a symmetric RFB using 0.5 mM THA‐{MV13} in 12 ml MeCN with 0.1 M THA‐BF4 at each side. **Supporting** **Table S1:** Melting behaviors of all samples. **Supporting** **Table S2:** Solubility calculation of IL samples in MeCN.

## Funding

This study was supported by Universität Ulm, Deutsche Forschungsgemeinschaft (DFG) (Grant 390874152).

## Conflicts of Interest

The authors declare no conflicts of interest.

## Supporting information

Supplementary Material

## Data Availability

The data that support the findings of this study are available from the corresponding author upon reasonable request.
