# Supplementary material for: Multiredox Polyoxovanadate‐Based Ionic Liquids for Nonaqueous Redox Flow Batteries
Source: ChemSusChem. 2026 Feb 22;19(4):e202502185. doi: 10.1002/cssc.202502185 (PMC12926725; doi:10.1002/cssc.202502185)
Supplement: Supplementary file 1 — Supplementary Material [file CSSC-19-e202502185-s001.pdf]

Electronic Supporting Information (ESI)

Multi-Redox Polyoxometalate-Based Ionic Liquids for Nonaqueous Redox Flow Batteries

Ke Wang,[a] Stefan Repp,[a] Moritz Remmers,[b] Boris Mashtakov,[b] Carsten Streb,* [b] Montaha Anjass*[a,c]

[a] M. Sc. K. Wang, Dr. S. Repp, Dr. M. Anjass
Institute of Inorganic Chemistry I
Ulm University
Albert-Einstein-Allee 11, 89081 Ulm, (Germany)
E-mail: montaha.anjass@uni-ulm.de

[b] M. Sc. M. Remmers, M. Sc. B. Mashtakov, Prof. Dr. C. Streb
Department of Chemistry
Johannes Gutenberg University Mainz
Duesbergweg 10-14, 55131 Mainz, (Germany)

[c] Dr. M. Anjass,
Department of Chemistry
University of Sharjah
P. O. Box 27272, Sharjah, United Arab Emirates University of Sharjah

Abstract: Redox-active ionic liquids (ILs) represent a promising class of energy carriers due to their intrinsic ionic conductivity, negligible volatility and electron-transfer capability. However, the design of ionic liquids capable of reversible multi-electron storage is still in its infancy. In this work, we report a family of mixed-valence polyoxovanadate-based ionic liquids (POV-ILs) obtained by combining the highly redox-active, mixed-valence cluster $(n\text{Bu}_4\text{N})_8[\text{V}_{14}\text{O}_{34}\text{Cl}][(\text{MgOH})\text{V}_{13}\text{O}_{33}\text{Cl}]$ with a series of bulky quaternary ammonium cations. Cation exchange transforms the solid precursor into liquid-like POV-ILs, dramatically enhancing solubility in organic solvents, such as acetonitrile, THF and glymes, making them ideal compounds for nonaqueous redox flow batteries (NRFBs). Electrochemical studies demonstrate that these POV-ILs retain the reversible multi-electron redox activity of the parent cluster across a wide potential window, enabling their use as symmetric electrolytes in nonaqueous redox flow batteries. Flow-cell demonstration confirms stable multi-electron cycling, with electrolyte remixing mitigating capacity fading. By integrating the redox versatility of POVs with the solubility and processability of ILs, this work establishes a new design strategy for redox-active electrolytes and highlights the promise of POV-ILs for next-generation, high-energy-density NRFBs.

Table of Contents

1. Chemicals and Instrumentation S2
2. Experimental Procedures S2-S3
   2.1 Synthesis S2-S3
   2.2 Electrochemical characterization S3
   2.3 Flow battery test S3
3. Results and Discussion S4-S15
   3.1. DSC analysis of TBA-**{MV$_{13}$}**, THA-**{MV$_{13}$}**, TOA-**{MV$_{13}$}**, TDA-**{MV$_{13}$}** S4
   3.2. ATR- FTIR spectroscopy of TBA-**{MV$_{13}$}**, THA-**{MV$_{13}$}**, TOA-**{MV$_{13}$}**, TDA-**{MV$_{13}$}** S5
   3.3. NMR analysis of TBA-**{MV$_{13}$}**, THA-**{MV$_{13}$}**, TOA-**{MV$_{13}$}**, TDA-**{MV$_{13}$}** S6-S9
   3.4. TGA of TBA-**{MV$_{13}$}**, THA-**{MV$_{13}$}**, TOA-**{MV$_{13}$}**, TDA-**{MV$_{13}$}** S10
   3.5. UV-vis spectroscopy of TBA-**{MV$_{13}$}**, THA-**{MV$_{13}$}**, TOA-**{MV$_{13}$}**, TDA-**{MV$_{13}$}** S11
   3.6. Solubility analysis of TBA-**{MV$_{13}$}**, THA-**{MV$_{13}$}**, TOA-**{MV$_{13}$}**, TDA-**{MV$_{13}$}** in solvents S12-S13
   3.7. Cycling performance of symmetric **{MV$_{13}$}**-ILs based RFBs S14
   3.8. References S14
   3.9. Author contributions S15

1. Chemicals and Instrumentation

Chemicals: Anhydrous MeCN (Acros Organics 99.9 % Extra Dry over molecular sieves, Acroseal®), tetrabutylammonium hexafluorophosphate (Sigma Aldrich, ≥ 99.0%), tetrahexylammonium tetrafluoroborate (Sigma Aldrich, ≥ 97.0%), tetraoctylammonium tetrafluoroborate (Sigma Aldrich, ≥ 97.0%), Ferrocene (Sigma Aldrich, 98%), AMI-7001 separator (Frontis Energy), Celgard® 2320 separator (Celgard), Carbon felt (SGL). tetrahexylammonium tetrafluoroborate and tetraoctylammonium tetrafluoroborate were recrystallized from pure ethanol and dried at 80 ^{o}C under vacuum before use. AMI-7001 and Celgard® 2320 were immersed in electrolyte solution for one day prior to battery tests.

Attenuated total reflection Fourier transform infrared spectroscopy (ATR-FTIR) was performed using a Bruker Vertex 70 Fourier transform infrared (FTIR) spectrometer (Bruker Optics, Ettlingen, Germany) equipped with a single-bounce ATR cell (GladiATR™, PIKE Technologies, Wisconsin, USA) and a liquid nitrogen cooled mercury cadmium telluride (MCT) detector. All spectra were recorded at room temperature in a spectral range from 4000 cm^{-1} to 400 cm^{-1} with a spectral resolution of 1 cm^{-1} averaging 64 scans.

UV/Vis Spectroscopy was recorded by a UV-VIS-NIR Spectrometer (JASCO 670). A quartz cuvette (path length = 1 cm) was used.

Thermogravimetric analysis (TGA) was carried out on a NETZSCH TG 209F1 analyzer at a heating rate of 10.0 K min^{-1} in a range between 25 and 700 °C under O_2/N_2 in an Al crucible.

Differential scanning calorimetry (DSC) was carried out by a differential scanning calorimeter (Mettler-Toledo DSC 2) under N_2 atmosphere with a heat rate of 10.0 K min^{-1}.

^{1}H nuclear magnetic resonance (1H-NMR) spectroscopy was recorded on a Bruker AVANCE Neo 400 MHz spectrometer at ambient temperature. ^{1}H-NMR spectra were measured at 400 MHz. Chemical shifts values (δ) are given in part per million (ppm) using residual solvent protons (δH = 2.50 ppm for DMSO-d_6).

2. Experimental Procedures

2.1 Synthesis:

Synthesis of (nBu$_4$N)$_4$[V$_{10}$O$_{26}$]: An orange suspension was prepared by dissolving 3.62 g vanadium (V) oxide (19.9 mmol) in 20 ml water and stirred at 60 ^{o}C. Subsequently, 7 ml triethylamine was added to the mixture with continuous stirring at 60 ^{o}C for 20 min. The resulting colorless/greyish solution was added to a solution of 18.9 g (nBu$_4$N)Br (58.6 mmol) in 100 mL acetone under stirring to obtain milky greyish precipitate. By the addition of a solution of 2.17 g $VOSO_4$ (13.3 mmol) in 5 mL water and 2 hours of stirring, black precipitate can be obtained. After filtration, the product was washed with water, ethanol and diethyl ether, consecutively.

Synthesis of $(nBu_4N)_8[V_{14}O_{34}Cl][(MgOH)V_{13}O_{33}Cl]$ = **TBA-{MV13}**: A greenish/black solution was obtained by stirring 1.003 g $(nBu_4N)_4[V_{10}O_{26}]$ (0.473 mmol, 1 eq.) and 0.101 g $MgCl_2$ (1.06 mmol, 2.2 eq.) in 25 ml acetonitrile at 75 ^oC for 4 h. After the filtration and subsequent diffusion of diethyl ether, the resulting crystals were washed consecutively with water, ethyl acetate and diethyl ether, and then dried. MW: 4499.31g/mol

Counter-cation exchange of $(nBu_4N)_8[V_{14}O_{34}Cl][(MgOH)V_{13}O_{33}Cl]$: A solution of $(nOctyl_4N)Br$/$(nDecyl_4N)Br$/$(nHeptyl_4N)Br$ (0.43 mmol, 3.95 eq.) in toluene (25 mL) was added into a mixture of 500 mg $(nBu_4N)_8[V_{14}O_{34}Cl][(MgOH)V_{13}O_{33}Cl]$ (0.11 mmol, 1 eq.) loaded onto water (25 mL). After stirred vigorously for 2 hours, the toluene phase was washed three times with water and evaporated. The final products were obtained after dried under vacuum at 80 °C.

Yield:
$(nHeptyl_4N)_8[V_{14}O_{34}Cl][(MgOH)V_{13}O_{33}Cl]$ = **THA-{MV13}** = 458.6 mg; MW = 5845.84 g/mol; 71 %
$(nOctyl_4N)_8[V_{14}O_{34}Cl][(MgOH)V_{13}O_{33}Cl]$ = **TOA-{MV13}** = 578.4 mg; MW = 6294.69 g/mol; 83 %
$(nDecyl_4N)_8[V_{14}O_{34}Cl][(MgOH)V_{13}O_{33}Cl]$ = **TDA-{MV13}** = 453.8 mg; MW = 7192.39 g/mol; 57 %

2.2 Electrochemical characterization:

Cyclic voltammetry (CV) and square wave voltammetry (SWV) was carried out using a Pine Research WaveDriver 200 electrochemical workstation. All experiments were performed in dry acetonitrile using 0.1 M supporting electrolyte. The solutions were purged with argon for at least 30 min to remove O_2 and kept under a slight positive argon pressure during the experiments. Cyclic voltammetry was performed in a standard three-electrode arrangement using a where carbon felt is used as working electrode, with platinum wire counter electrode, and silver wire in a glass frit containing electrolyte solution, as quasi-reference electrode. Concentration of $\{MV_{13}\}$ clusters were used at 0.5 mM. Ferrocene was used as internal reference standard.

2.3 Flow battery test

The charge-discharge battery tests were performed using galvanostatic method by a symmetric RFB cell in an Argon-filled glove box. When the concentration of **{MV13}**-ILs was 0.5 mM in the battery test; the positive and negative half-cells were separated by two pieces of microporous separator (Celgard® 2320; 20 μm thickness for single piece, 39% porosity). Prior to use, the separators were immersed into the blank electrolyte solution for 1 day. Carbon felts were used as-received and the electrode area is 7.29 cm^2 (2.7 cm * 2.7 cm). The electrodes were clamped tightly on the both sides of the separators for a 'zero-gap' cell assembly. Graphite plates with serpentine flow field were used as current collectors. PTFE and EPDM spacer were used for the cell sealing. 12 mL of electrolytes for the positive and negative sides were identical: 0.5 mM redox material, 0.1 M supporting salt, MeCN as solvent. During the battery operation, electrolytes were circulated in a flow rate of 40 ml/min between the electrolyte tanks and the reaction cell. Charging current density and discharging current density were 0.04 mA cm^{-2} and 0.02 mA cm^{-2}, respectively. The cut-off voltage for charging and discharging process were 0.7 V and 0.02 V, respectively.

When the concentration of **{TOA-V13}** was 5 mM in the battery test, a piece of AMI-7001 was used as the separator, and the electrolyte volume was 10 ml for each half cell. Charging current density and discharging current density were 0.02 mA cm^{-2}. The cut-off voltage for charging and discharging process were 1.32 V and 0 V, respectively.

When the concentration of **{TOA-V13}** was 60 mM in the battery test in the static mode, a piece of AMI-7001 was used as the separator, and the electrolyte volume was 0.25 ml for each half cell. Charging current density and discharging current density were 0.02 mA cm^{-2}. The cut-off voltage for charging and discharging process were 1.5 V and 0 V, respectively.

Results and Discussion

1. Differential scanning calorimetry (DSC) analysis

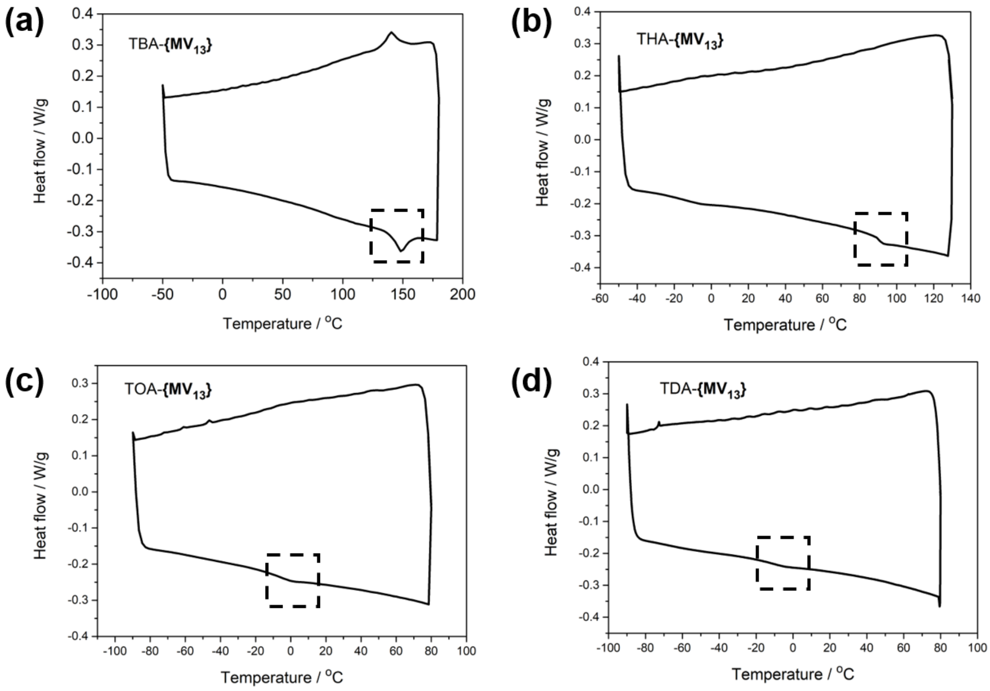

Figure S1. DSC analysis of (a) TBA-**{MV$_{13}$}**, (b) THA-**{MV$_{13}$}**, (c) TOA-**{MV$_{13}$}**, and (d) TDA-**{MV$_{13}$}**, under N_2 atmosphere with the heating rate of 10.0 K min^{-1}.

Table 1. Melting behaviors of all samples

Sample	Thermal event	Temperature (°C)
**TBA-{MV$_{13}$}**	melting peak	153
**THA-{MV$_{13}$}**	glass transition	91
**TOA-{MV$_{13}$}**	glass transition	-5
**TDA-{MV$_{13}$}**	glass transition	-12

2. IR spectroscopy

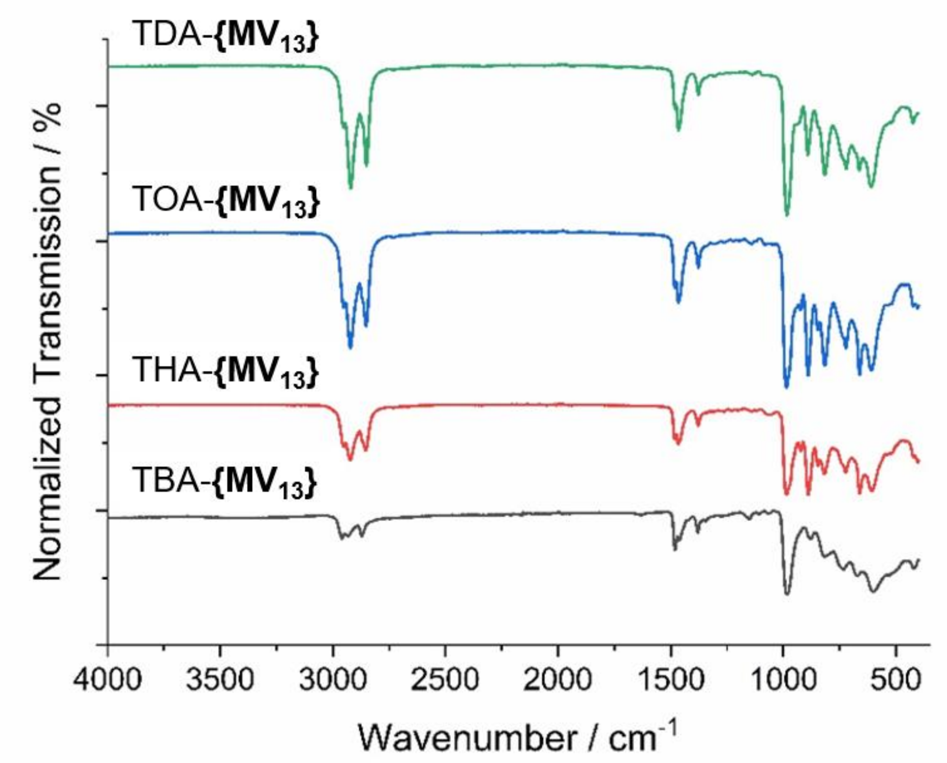

Figure S2. ATR-FTIR spectra of TBA-**{MV$_{13}$}**, THA-**{MV$_{13}$}**, TOA-**{MV$_{13}$}**, TDA-**{MV$_{13}$}** normalized Transmission.

3. NMR analysis

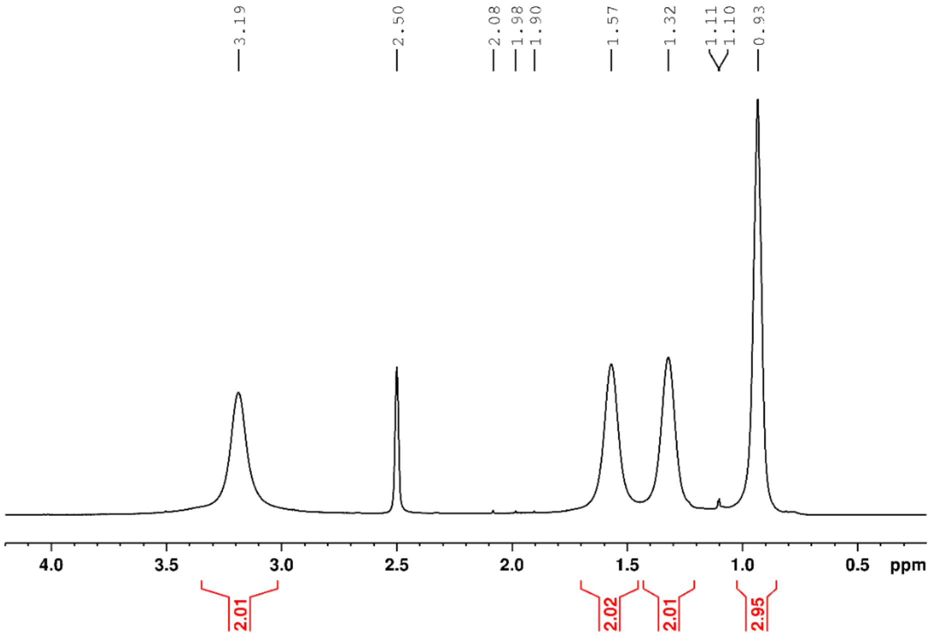

Figure S3. ^{1}H-NMR of **TBA-{MV$_{13}$}**. Conditions: solvent: DMSO-d_6, 400 MHz, 16 scans. Signal assignments: δ (ppm) = 3.19 (m, 2 H, 1 x CH_2, nButyl$_4$N$^+$); 2.50 (s, DMSO); 2.08 (impurity); 1.98 (impurity); 1.90 (impurity); 1.57 (m, 2 H, 1 x CH_2, nButyl$_4$N$^+$); 1.32 (m, 2 H, 1 x CH_2, nButyl$_4$N$^+$); 1.10 (impurity); 0.93 (m, 3 H, 1 x CH_3, nButyl$_4$N$^+$).

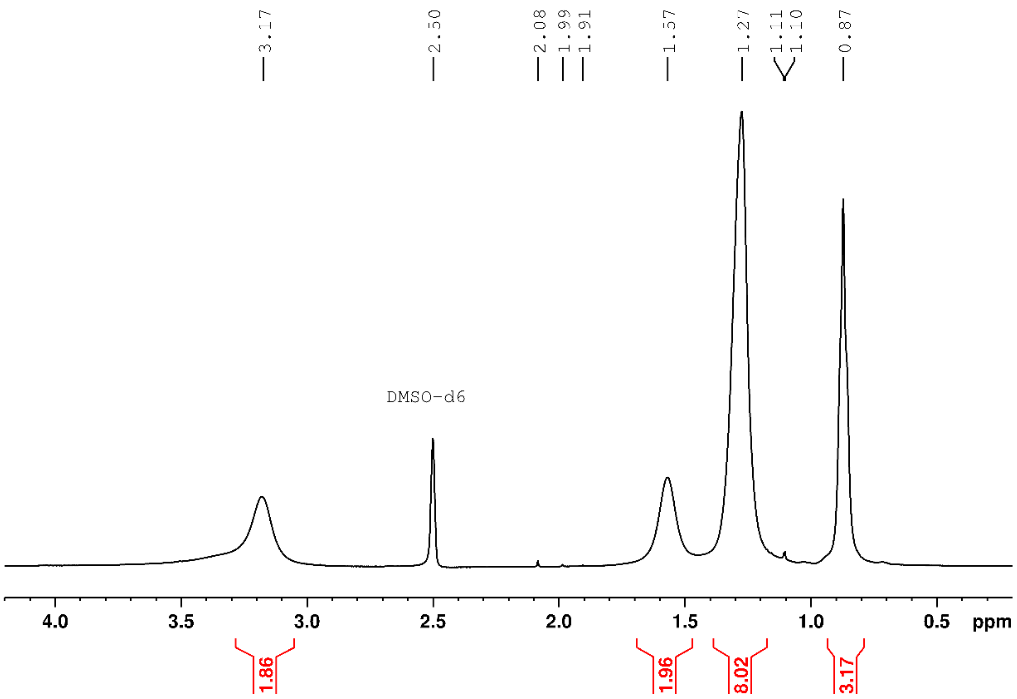

Figure S4. ¹H-NMR of THA-{MV_{13}}. Conditions: solvent: DMSO-d_6, 400 MHz, 16 scans. Signal assignments: δ (ppm) = 3.17(m, 2 H, 1 x CH_2, $nHeptyl_4N^+$); 2.50 (s, DMSO); 2.08 (impurity); 1.98 (impurity); 1.90 (impurity); 1.57 (m, 2 H, 1 x CH_2, $nHeptyl_4N^+$); 1.27 (m, 8 H, 4 x CH_2, $nHeptyl_4N^+$); 1.10 (impurity); 0.93 (m, 3 H, 1 x CH_3, $nHeptyl_4N^+$).

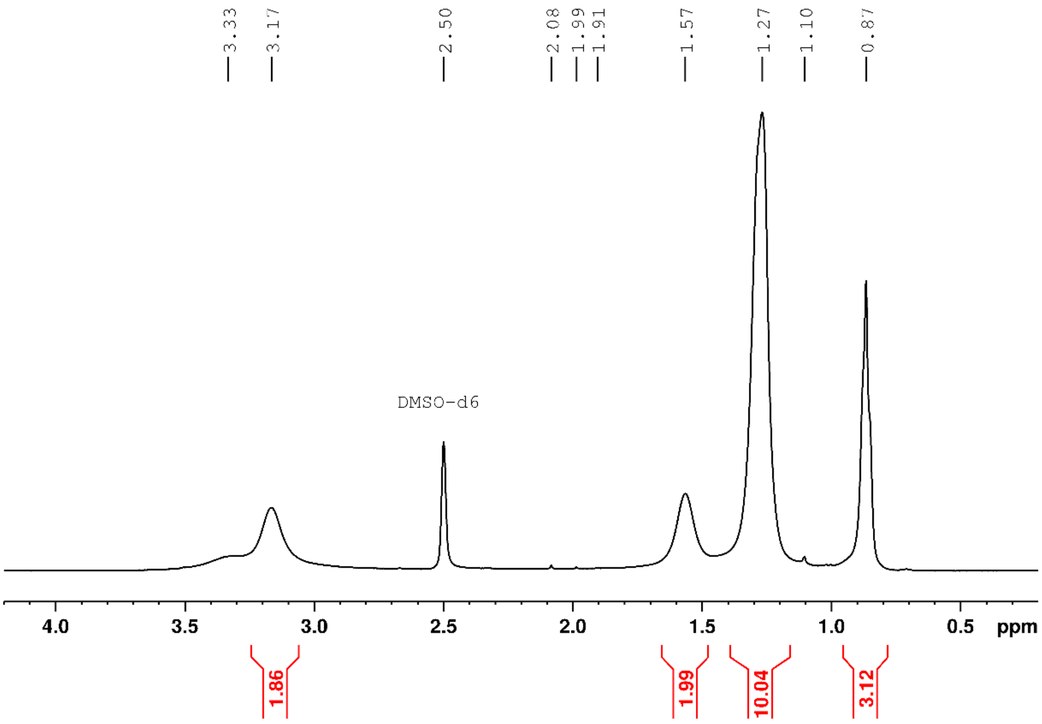

Figure S5. ¹H-NMR of TOA-{MV_{13}}. Conditions: solvent: DMSO-d_6, 400 MHz, 16 scans. Signal assignments: δ (ppm) = 3.33 (water); 3.17(m, 2 H, 1 x CH_2, $nOctyl_4N^+$); 2.50 (s, DMSO); 2.08 (impurity); 1.99 (impurity); 1.91 (impurity); 1.57 (m, 2 H, 1 x CH_2, $nOctyl_4N^+$); 1.27 (m, 10 H, 5 x CH_2, $nOctyl_4N^+$); 1.10 (impurity); 0.87 (m, 3 H, 1 x CH_3, $nOctyl_4N^+$

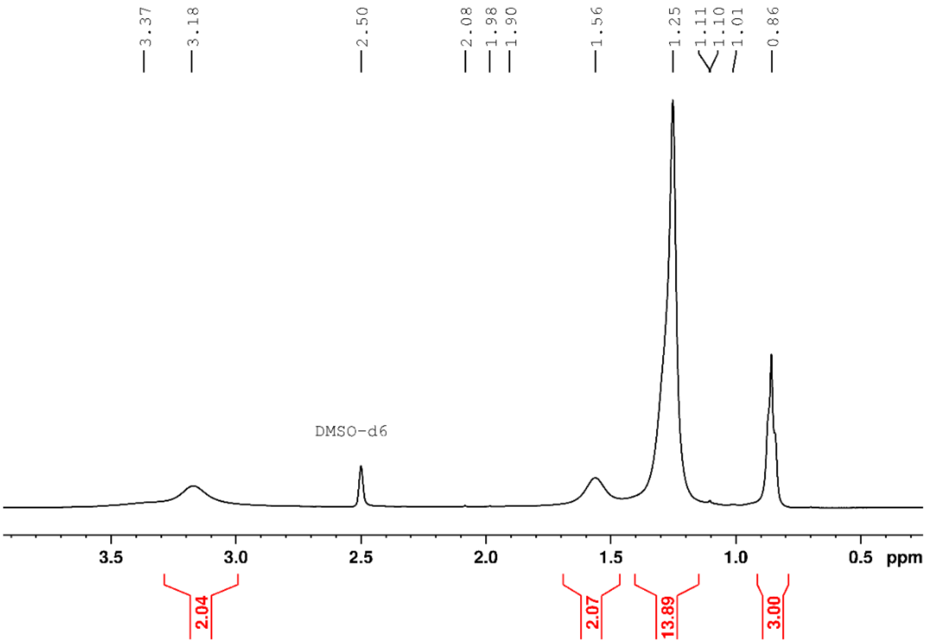

Figure S6. ^{1}H-NMR of **TDA-{MV$_{13}$}**. Conditions: solvent: DMSO-d$_6$, 400 MHz, 16 scans. Signal assignments: δ (ppm) = 3.37 (water); 3.18(m, 2 H, 1 x CH$_2$, nDecyl$_4$N$^+$); 2.50 (s, DMSO); 2.08 (impurity); 1.98 (impurity); 1.90 (impurity); 1.56 (m, 2 H, 1 x CH$_2$, nDecyl$_4$N$^+$); 1.25 (m, 14 H, 7 x CH$_2$, nDecyl$_4$N$^+$); 1.10 (impurity); 1.01 (impurity); 0.86 (m, 3 H, 1 x CH$_3$, nDecyl$_4$N$^+$).

4. TGA

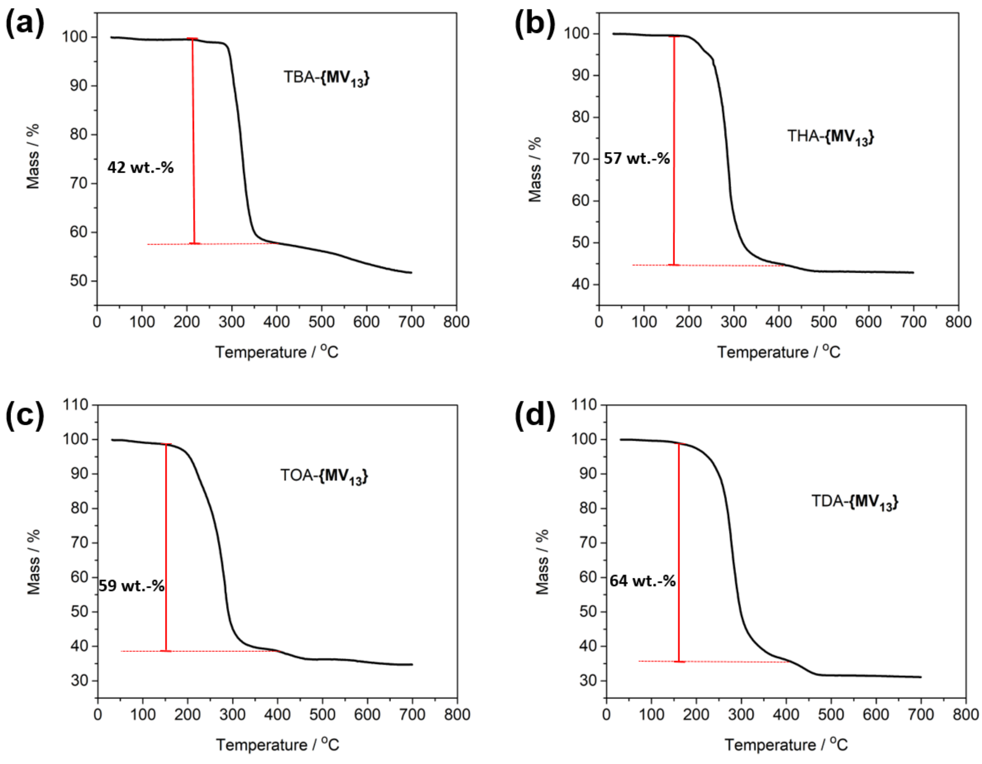

Figure S7. TGA tests of TBA-**{MV$_{13}$}**, THA-**{MV$_{13}$}**, TOA-**{MV$_{13}$}**, TDA-**{MV$_{13}$}**, respectively, at a heating rate of 10.0 K min^{-1} in a range between 25 and 700 °C under O_2/N_2. which showed weight loss of 42 wt.-% (calcd.: 43 wt.-%), 57 wt.-% (calcd.: 56.2 wt.-%), 59 wt.-% (calcd.: 59.3 wt.-%), and 64 wt.-% (calcd.: 64.4 wt.-%) for TBA-**{MV$_{13}$}**, THA-**{MV$_{13}$}**, TOA-**{MV$_{13}$}**, TDA-**{MV$_{13}$}**, respectively.

5. UV-vis spectroscopy

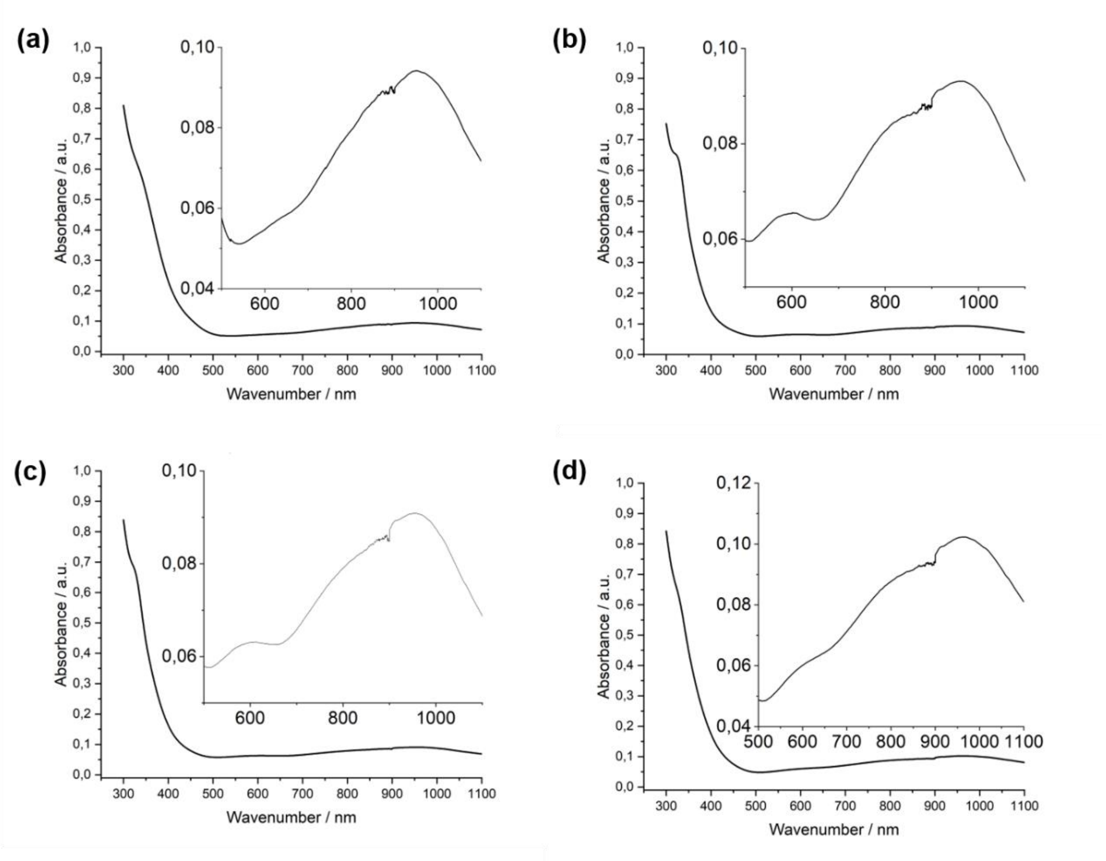

Figure S8. UV-Vis spectra of (a) 0.025 mMol **TBA-{MV$_{13}$}**, (b) 0.025 mMol **THA-{MV$_{13}$}**, (c) 0.025 mMol **TOA-{MV$_{13}$}**, (d) 0.025 mMol **TDA-{MV$_{13}$}**.

6. TBA-{MV$_{13}$} and {MV$_{13}$}-ILs in organic solvents

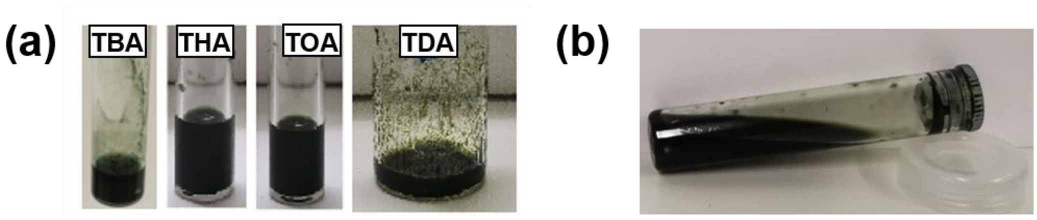

Figure S9. (a) 30 mM **TBA-{MV$_{13}$}** in MeCN, 60 mM **THA-{MV$_{13}$}** in MeCN, 60 mM **TOA-{MV$_{13}$}** in MeCN, 60 mM **TDA-{MV$_{13}$}** in MeCN, respectively; (b) approximate 130 mM **TOA-{MV$_{13}$}** in MeCN.

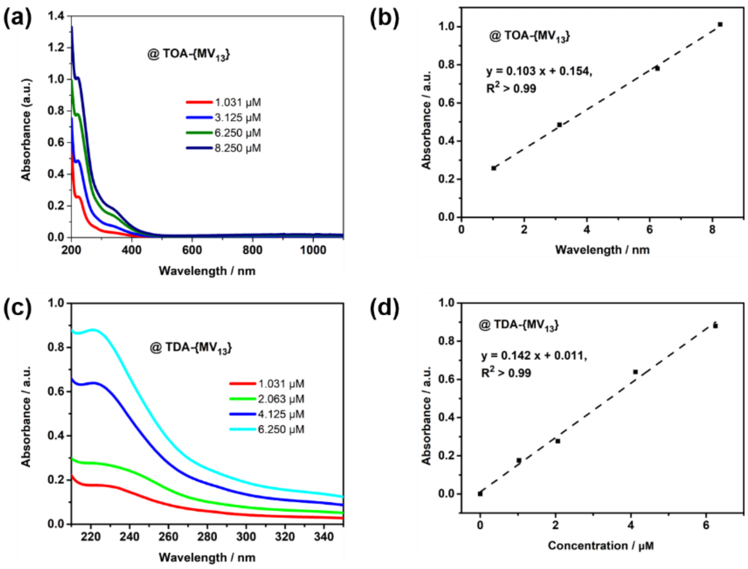

Figure S10. (a) Absorbance spectra of **TOA-{MV$_{13}$}** in MeCN; (b) Beer Lambert plot of absorbance of **TOA-{MV$_{13}$}** in MeCN at 221 nm; (c) absorbance spectra of **TDA-{MV$_{13}$}** in MeCN; (d) Beer Lambert plot of absorbance of **TDA-{MV$_{13}$}** in MeCN at 221 nm.

Table 2. Solubility calculation of IL samples in MeCN.

Sample	Absorbance (221 nm)	Dilute concentration (μM)	Dilution times	Saturated concentration (mM)
TOA-**{MV$_{13}$}**	0.581	4.150	32000	132.8
TDA-**{MV$_{13}$}**	0.278	1.877	16000	30.032

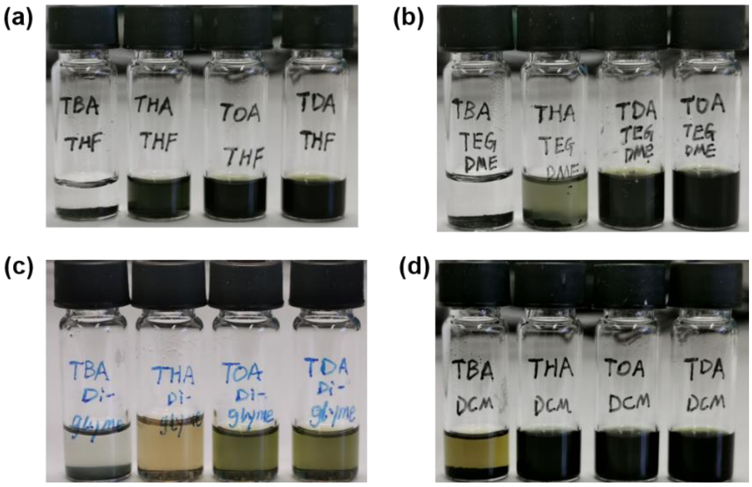

Figure S11. The digital photos of **TBA-{MV$_{13}$}** and **{MV$_{13}$}-ILs** in (a) THF; (b) TEGDME; (c) Digylme; (d) DCM.

7. Cycling performance of symmetric {MV_{13}}-ILs based RFBs

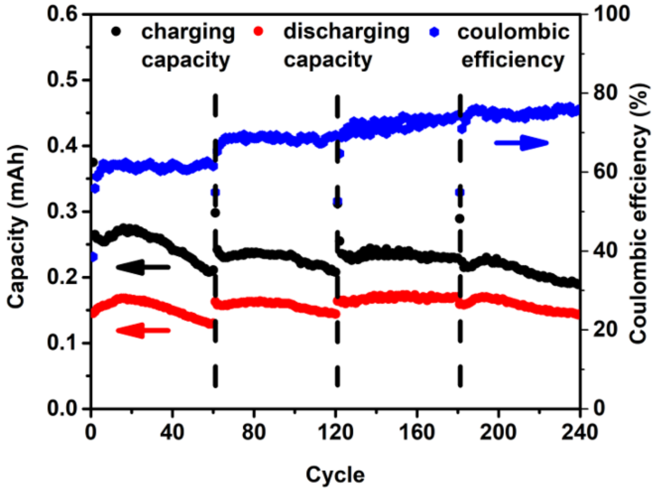

Figure S12. Cycling performance of a symmetric RFB using 0.5 mM **TOA-{MV_{13}}** in 12 ml MeCN with 0.1 M $THABF_4$ at each side.

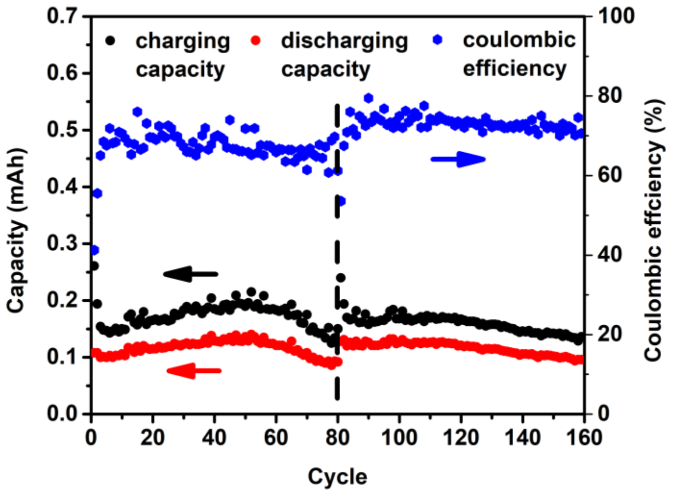

Figure S13. Cycling performance of a symmetric RFB using 0.5 mM **THA-{MV_{13}}** in 12 ml MeCN with 0.1 M THA-BF_4 at each side.

Reference

1. L. E. VanGelder, A. M. Kosswattaarachchi, P. L. Forrestel, T. R. Cook, E. M. Matson, *Chem. Sci*. **2018**, *9*, 1692-1699.
2. L. E. VanGelder, B. E. Petel, O. Nachtigall, G. Martinez, W. W. Brennessel, E. M. Matson, *ChemSusChem* **2018**, *11*, 4139-4149.
3. S. Greiner, B. Schwarz, M. Ringenberg, M. Dürr, I. Ivanovic-Burmazovic, M. Fichtner, M. Anjass, C. Streb, *Chem. Sci.* **2020**, *11*, 4450-4455.
4. S. Greiner, B. Schwarz, C. Streb, M. Anjass, *Chem. Eur. J*. **2021**, *27*, 13435-13441.
